# Treatment of refractory catatonic schizophrenia with low dose aripiprazole

**DOI:** 10.1186/1744-859X-11-12

**Published:** 2012-05-03

**Authors:** Tsuyoshi Sasaki, Tasuku Hashimoto, Tomihisa Niitsu, Nobuhisa Kanahara, Masaomi Iyo

**Affiliations:** 1 Department of Child Psychiatry, Chiba University Hospital, Inohana 1-8-1, Chiba, 260-8670, Japan; 2 Department of Psychiatry, Chiba University Graduate School of Medicine, Inohana 1-8-1, Chiba, 260-8670, Japan; 3 Research Center for Child Mental Development, Chiba University Graduate School of Medicine, Inohana 1-8-1, Chiba, 260-8670, Japan

## Abstract

This case is of 54-year-old female with catatonic schizophrenia, characterized by treatment resistance to the pharmacotherapy with olanzapine, risperidone, flunitrazepam, and ECT. Olanzapine and risperidone and flunitrazepam did not improve her catatonic and psychotic symptoms, and induced the extrapyramidal symptoms. The effects of ECT did not continue even for a month. However, the treatment with low-dose aripiprazole dramatically improved the patient’s psychotic symptoms and extrapyramidal symptoms. The mechanisms underlying the effects of low-dose aripiprazole in this case remain unclear, but unlike other antipsychotics, aripiprazole is a dopamine D2 partial agonist. In this regard, our results suggest that aripiprazole has numerous advantages, especially in cases of stuporous catatonia and a defective general status.

## Background

Catatonia, which is characterized by motoric immobility such as catalepsy or stupor, mutism, negativism, is shown in about 10-15% of patients with schizophrenia [1]. Published works to date demonstrate variable treatment with benzodiazepines, electroconvulsive therapy (ECT), N-Methyl-D-Asparte (NMDA) antagonists, and antipsychotics, even the atypical, which remain discussed, because of worsening in symptomatology and increasing the risk of inducing neuroleptic malignant symdrome [2-4]. Aripiprazole, a dopamine D2 receptor partial agonist, is different from other atypical antipsychotics, which are common profile of D2 receptor antagonist.

We present a patient of catatonic schizophrenia, which was markedly improved on low-dose aripiprazole, after failing to respond to olanzapine, risperidone, and ECT.

## Case presentation

A 54-year-old woman began to experience auditory hallucinations, including a voice admonishing her to deny herself, and delusions of persecution and reference, and to withdraw socially. She gradually entered a catatonic stupor with insomnia and anorexia. Her mother took her to a mental clinic, where she was diagnosed with catatonic-type schizophrenia. She was prescribed risperidone 1 mg/day and flunitrazepam 1 mg/day for approximately 4 months, but her withdrawal and stupor state did not improve, and she was referred to another hospital at 55 years of age. No abnormalities were found in her general laboratory examinations or brain CT. She reported having consistent pathological experiences, such as hallucination and delusions, and her diagnosis was confirmed as catatonic-type schizophrenia according to the DSM-IVcriteria. We discontinued her previous medications and prescribed olanzapine 20 mg/day and flunitrazepam 1 mg/day for approximately 2 weeks. However, her catatonic stupor with anorexia and severe extrapyramidal symptoms was not improved. She was referred to a general hospital, and began to receive electroconvulsive therapy (ECT). The ECT treatment was enforced twice for six weeks per week (total 12 times). The treatment with ECT and risperidone 3 mg/day and flunitrazepam 2 mg/day almost completely eliminated her psychotic symptoms, and she was again able to take meals by herself, but a high level of extrapyramidal symptoms remained. She was transferred to our hospital. After another month of ECT, however, she began to experience auditory hallucinations, and delusions of persecution and reference, along with stupor with insomnia and anorexia again. We changed her drug regimen from risperidone and flunitrazepam to only aripiprazole 3 mg/day. Two weeks later, her catatonic stupor and psychotic symptoms were dramatically improved without extrapyramidal symptoms. She was again able to take meals alone and was able to go shopping with her mother. She is now 59 years old and has been treated with only 3 mg/day aripiprazole for 48 months. She has no recrudescence of psychotic symptoms or extrapyramidal symptoms, and continues to help her mother with the housework.

## Discussion

This case is of 54-year-old female with catatonic schizophrenia, characterized by treatment resistance to the pharmacotherapy with olanzapine and risperidone and flunitrazepam, and ECT. To our knowledge, this is the first published report to show that the treatment-resistant schizophrenic patient with the catatonic subtype was improved, using ultra-low-dose aripiprazole. In this case, olanzapine and risperidone and flunitrazepam did not improve her catatonic and psychotic symptoms, and induced the extrapyramidal symptoms. The effects of ECT did not continue even for a month. However, the treatment with low-dose aripiprazole dramatically improved the patient’s psychotic symptoms and extrapyramidal symptoms. Although it has been reported that atypical antipsychotics and benzodiazepines were effective for stuporous catatonia [5], olanzapine and risperidone ,and flunitrazepam did not improve the psychotic symptoms of our patient; moreover, they exacerbated her extrapyramidal symptoms. Although benzodiazepines, such as lorazepam, are the first choice for the catatonic features as well as ECT [5], and their high-doses are frequently used, benzodiazepines might cause respiratory arrest. So we were not able to used for it. The mechanisms underlying the effects of low-dose aripiprazole in this case remain unclear. Recently Vörös et al and Kirino reported that aripiprazole is effective for catatonic stupor [6,7]. Also it has been reported that ECT-aripiprazole combination therapy has an excellent safety profile and therapeutic efficacy [8,9]. The present case responded to ECT but began to relapse one month after the course of ECT. So without the effects of ECT, it would be uncertain whether aripiprazole alone could have resolved her long-term stupor state. In this regard, our results suggest that aripiprazole might have advantages, especially in cases of stuporous catatonia and a defective general status like the present case.

## Conclusion

We reported here a case of successful treatment using low-dose aripiprazole monotherapy for a female patient after the ECT in cases of stuporous catatonia and a defective general status.

## Consent

Written informed consent was obtained from the patient for publication of this case report and any accompanying images. A copy of the written consent is available for review by the Editor-in-Chief of this journal.

## Competing interests

The authors declare that they have no competing interests.

## Authors’ contributions

TS, TH, TN and NK wrote the manuscript. MI is the principal investigator of this study. All authors read and approved the final manuscript.
